# Residual tissue repositories as a resource for population-based cancer proteomic studies

**DOI:** 10.1186/s12014-018-9202-4

**Published:** 2018-08-03

**Authors:** Paul D. Piehowski, Vladislav A. Petyuk, Ryan L. Sontag, Marina A. Gritsenko, Karl K. Weitz, Thomas L. Fillmore, Jamie Moon, Hala Makhlouf, Rodrigo F. Chuaqui, Emily S. Boja, Henry Rodriguez, Jerry S. H. Lee, Richard D. Smith, Danielle M. Carrick, Tao Liu, Karin D. Rodland

**Affiliations:** 1 Biological Sciences Division, Pacific Northwest National Laboratory, Richland, WA 99354 USA; 2 Division of Cancer Treatment and Diagnosis, National Cancer Institute, Rockville, MD 20850 USA; 3 Office of Cancer Clinical Proteomics Research, National Cancer Institute, Bethesda, MD 20892 USA; 4 Center for Strategic Scientific Initiatives, National Cancer Institute, Bethesda, MD 20892 USA; 5 Division of Cancer Control and Population Sciences, National Cancer Institute, Rockville, MD 20850 USA

**Keywords:** Proteomics, Phosphoproteomics, Tandem mass tags, Formalin fixed paraffin embedded, Surveillance, epidemiology and end results

## Abstract

**Background:**

Mass spectrometry-based proteomics has become a powerful tool for the identification and quantification of proteins from a wide variety of biological specimens. To date, the majority of studies utilizing tissue samples have been carried out on prospectively collected fresh frozen or optimal cutting temperature (OCT) embedded specimens. However, such specimens are often difficult to obtain, in limited in supply, and clinical information and outcomes on patients are inherently delayed as compared to banked samples. Annotated formalin fixed, paraffin embedded (FFPE) tumor tissue specimens are available for research use from a variety of tissue banks, such as from the surveillance, epidemiology and end results (SEER) registries’ residual tissue repositories. Given the wealth of outcomes information associated with such samples, the reuse of archived FFPE blocks for deep proteomic characterization with mass spectrometry technologies would provide a valuable resource for population-based cancer studies. Further, due to the widespread availability of FFPE specimens, validation of specimen integrity opens the possibility for thousands of studies that can be conducted worldwide.

**Methods:**

To examine the suitability of the SEER repository tissues for proteomic and phosphoproteomic analysis, we analyzed 60 SEER patient samples, with time in storage ranging from 7 to 32 years; 60 samples with expression proteomics and 18 with phosphoproteomics, using isobaric labeling. Linear modeling and gene set enrichment analysis was used to evaluate the impacts of collection site and storage time.

**Results:**

All samples, regardless of age, yielded suitable protein mass after extraction for expression analysis and 18 samples yielded sufficient mass for phosphopeptide analysis. Although peptide, protein, and phosphopeptide identifications were reduced by 50, 20 and 76% respectively, from comparable OCT specimens, we found no statistically significant differences in protein quantitation correlating with collection site or specimen age. GSEA analysis of GO-term level measurements of protein abundance differences between FFPE and OCT embedded specimens suggest that the formalin fixation process may alter representation of protein categories in the resulting dataset.

**Conclusions:**

These studies demonstrate that residual FFPE tissue specimens, of varying age and collection site, are a promising source of protein for proteomic investigations if paired with rigorously verified mass spectrometry workflows.

**Electronic supplementary material:**

The online version of this article (10.1186/s12014-018-9202-4) contains supplementary material, which is available to authorized users.

## Background

The ability to develop targeted therapies for cancer and other diseases depends heavily on the ability to identify functional changes that not only distinguish between the healthy and diseased states, but that also reflect clinical outcomes. However, for many diseases of interest, the time period between initial diagnosis and significant clinical outcomes, including disease recurrence or death, is sufficiently long that prospective clinical trials involving fit-for-purpose tissue samples are extraordinarily expensive. In this regard, the availability of archived formalin fixed, paraffin embedded (FFPE) tissue blocks, obtained at the time of initial diagnosis (i.e., residual tissue remaining after pathology review for diagnosis) and archived with appropriate metadata and clinical follow-up, represents an invaluable resource for studies on prognostic and predictive markers of cancer development and progression.

There are 18 surveillance, epidemiology, and end results (SEER) cancer registries that cover approximately 28% of the United States population, providing high quality demographic, clinical, pathologic, and survival data. In three of the SEER registries (Los Angeles, Iowa, Hawaii), annotated FFPE tumor tissue specimens are available for research use through established residual tissue repositories (RTR) [1, 2], with FFPE blocks dating back over 30 years available for research purposes upon request. Archived FFPE tissues from SEER have been a source of biospecimens linked to demographic data, tumor stage information, survival data, and other electronic records for cancer researchers for decades [2]. Further, development of population-based biospecimen research capacity using SEER, and other similar residual tissue repositories, offers opportunities for unbiased sampling and collection of robust samples providing crucial outcomes data to aid in the interpretation of deep molecular analyses, such as those provided by next-generation sequencing (NGS) and proteomic methods.

However, the utility of FFPE blocks collected and processed in routine but non-uniform clinical settings, and ultimately stored for many years in various settings, needs to be evaluated in the context of mass spectrometry (MS)-based proteomics to confirm the appropriateness of these residual tissue resources for both data dependent analysis (DDA) and data independent analysis (DIA) [3]. Whole exome sequencing (WES) analysis of SEER FFPE blocks indicated that a very high percentage of SEER FFPE blocks stored between 3 and 32 years provided sufficient quantity and quality of DNA for WES [4]. The suitability of archival FFPE specimens, prepared in numerous pathology labs under varying laboratory conditions and stored for varying lengths of time, remains to be established for MS-based proteomics.

The main challenge for proteomic analysis of FFPE specimens is the inherent and significant cross-linking between proteins and other molecules in the tissue through Schiff base formation [5]. This cross-linking hinders efficient and reproducible extraction of proteins, and the presence of chemical modifications impedes peptide identification [6]. Thus, effective reversal of protein modifications is vital to successful quantitative analyses. Due to the considerable interest in this type of biological specimen, significant effort has been directed at addressing these challenges in FFPE samples [7, 8], and a number of effective protein extraction strategies have been previously reported in the literature [9–14]. These methods employ strong detergents like SDS [9], sample boiling [9–14], denaturing solvents [11], and high concentrations of primary amine containing buffers [13] or combinations thereof, to aid in solubilization and to reverse crosslinking of proteins. For our analysis we used a 2,2,2-trifluoroethanol (TFE)-based extraction protocol [11] coupled with xylene for deparaffinization, as we found that it performed best in our hands and produced sufficient protein yields (> 100 µg) for our proteomic pipeline.

In addition to challenges in protein extraction, it is also important to consider the potential impacts of pre-analytical factors on the quality and consistency of FFPE specimens [15, 16]. For example, a lack of standardization of FFPE protocols, such as formalin fixation time, may result in varying degrees of chemical cross-linking [17]. Other related issues of potential significance include the time from harvesting to fixation and the quality of the tissue harvested, as well as specimen storage time, temperature, light exposures and more [18]. The impact of storage time was evaluated by Craven and co-workers using label-free proteomics, and it was found that storage times up to 10 years had no measurable impact on protein abundances [19]. Here we expand our investigation to specimens as old as 32 years, and include the use of TMT labeling and high pH fractionation which greatly expands the depth of proteomic coverage.

Because de-regulation of protein phosphorylation is a recognized hallmark of cancer and disease [20], we were particularly interested in evaluating phosphopeptide information available from the SEER specimens. A number of studies using immunohistochemistry, as well as LC–MS based phosphoproteomics, suggest that valuable phosphorylation information is retained in FFPE specimens [21–25]. In this study, we investigated whether protein extracted from FFPE tissues archived in SEER RTRs is of sufficient quantity and quality for quantitative MS-based proteomic and phosphoproteomic analysis, and examined the effects of storage time on tumor proteomes and phosphoproteomes, to determine the potential utility of FFPE blocks collected over clinically significant time periods. Because our study used existing archived FFPE blocks in the SEER RTRs, we were able to use a large sample cohort and linear modeling techniques to assess the utility of commonly available archived FFPE samples [26], even though we could not follow the same FFPE sample over an extended storage time.

## Methods

### Subject/specimen selection

Fifty-nine of the 60 FFPE tissue sections used in this study were from the same SEER cases used in a previous study of whole exome sequencing (WES), with subject and specimen selection information described previously [4]. Fixation times/conditions and storage conditions are unknown, as specimens were retrospectively collected by the RTRs from multiple medical facilities and pathology labs within each of the three catchment areas. Tissues were from high-grade serous ovarian adenocarcinomas (ICD-O-3 Topography code: C56.9; Morphology codes: 8441/3, 8460/3, 8461/3), and storage time ranged from 7 to 32 years (Table 1) based on when tissue was resected. Each SEER registry also conducted a pathology review of lead and trail sections flanking the five sections from each tissue block used for proteomics, to determine whether tissue was consistent with the selection criteria (high-grade serous ovarian adenocarcinoma, ≥ 50% of cells with nuclei consistent with malignant cells, and ≤ 50% of cells were necrotic); approximately 30 cases from each registry were reviewed to ultimately select 20 cases that met study criteria. The NCI-conducted pathology review verified that the majority of tissues met desired selection criteria; 77% of the tissues sent had ≥ 50% of cells with nuclei consistent with malignant cells and 98% had ≤ 50% necrotic cells. For each case identified as meeting the study criteria, five 10-µm sections were placed in a sterile tube and sent to the Pacific Northwest National Laboratory (PNNL). Two of the SEER registries also supplied information allowing the dimensions of the tumor tissue on the slides used for pathology review to be determined, and this information was used to calculate peptide yield per tumor volume (mm^3^).

**Table 1:** FFPE specimens selected for analysis

	Specimen time in storage				
	3–12 years	13–22 years	23–32 years	Age not provided	Total
RTR site 1	8	9	3	0	20
RTR site 2	4	11	5	0	20
RTR site 3	1	11	7	1	20
Total	13	31	15	1	60

### FFPE sample processing for expression (global) proteomics using TMT 10-plex labeling

Upon receipt of the specimens, tissue quality was assessed by conducting gross QC checks of tissue sections for damaged FFPE curls. Samples were then randomized into batches of 20 for ease of sample preparation and to remove correlations with confounding technical factors. Tissue curls (5 curls per sample) were transferred to a 2.0 mL screw-top tube and de-paraffinized twice, using 500 µL xylenes with end-over-end rotation for 5 min at room temperature. The process was repeated with absolute ethanol and 80% ethanol in water, before drying in a Speed-Vac concentrator for 10 min.

A 200 µL aliquot of 50:50 TFE:600 mM Tris was added to the de-paraffinized tissue, followed by 200 µL 50 mM ammonium bicarbonate (ABC). Two µL of phosphatase inhibitor cocktails 2 and 3 (Sigma-Aldrich) and Halt protease inhibitor (ThermoFisher) were added, and the sample was incubated at 99 °C for 90 min with shaking at 1000 rpm. The supernatant was assayed for protein concentration by BCA assay (ThermoFisher) before the entire sample was reduced with 5 mM dithiothreitol (DTT) (Sigma) for 1 h at 37 °C. Reduced cysteines were alkylated with 40 mM iodoacetamide (IAA) (Sigma-Aldrich) for 1 h at 37 °C in the dark. The sample was diluted fivefold with 50 mM ABC buffer and trypsin was added at a 1:50 enzyme:substrate ratio, followed by incubation overnight (~ 16 h) at 37 °C. Each sample was centrifuged at 10,000 rpm for 10 min to pellet remaining tissue debris. The resulting peptides were desalted using C18 solid phase extraction (SPE) cartridges (Discovery C18, Supelco). Forty µg of each sample was prepared for TMT isobaric labeling (ThermoFisher, Rochester, NY) by reconstituting the peptides with 100 µL of 100 mM triethylammonium bicarbonate (TEAB). A 10 µg aliquot of each sample was combined for a reference pool (30 µg each), used as a common reference for normalization, and labeled by the TMT 131 reagent while the remaining 30 µg per sample was assigned randomly to one of the other nine channels in a total of seven separate TMT10-plex experiments (Additional file 1: Table S1). This pooled reference sample was used as a common denominator in each TMT-10 plex, allowing precise comparison of relative protein abundances across the entire sample set [27]. Each isobaric tag aliquot was dissolved in 41 µL anhydrous acetonitrile by vortexing for 5 min, and added to each sample. After incubation at room temperature for 1 h, the reaction was quenched by addition of 8 µL of 5% hydroxylamine in 100 mM TEAB with incubation at room temperature for 15 min. Each sample in the 10-plex experiment was combined and concentrated in a Speed-Vac before undergoing another C18 SPE cleanup. Each 10-plex experiment was fractioned into 96 fractions with by high pH reversed phase separation, followed by concatenation into 24 fractions for MS analysis as described previously [28].

### Phosphopeptide enrichment using IMAC

Eighteen samples covering a broad sample storage time and with sufficient peptide yield were also selected for phosphoproteomics analysis using TMT. Similar to the expression proteomics analysis, a pooled reference was created by combining a 40-µg aliquot from each of the 18 samples, (Additional file 2: Table S2). Magnetic Fe^3+^-NTA-agarose beads were freshly prepared for phosphopeptide enrichment using the Ni-NTA-agarose beads (QIAGEN, #36111) [27]. For each individual sample (including the pooled reference), 300 µg peptides were reconstituted in 600 μL IMAC binding/wash buffer [80% acetonitrile, 0.1% formic acid (FA)] and incubated with 150 μL of the 5% bead suspension for 30 min at RT in a thermomixer with constant shaking at 800 rpm. After incubation, the beads were washed 4 times each with 600 μL of wash buffer to remove any non-specific binding. Phosphopeptides were eluted from the beads using 180 μL of 500 mM K_2_HPO_4_ (pH 7.0) directly on C18 Stage tips and eluted from C18 material with 100 μL 50% ACN, 0.1% FA. Samples were dried in a Speed-Vac concentrator, and were reconstituted in 10 μL of 50 mM HEPES, pH 8.5 for TMT-10 labeling [29]. TMT reagents were rehydrated in 40 μL anhydrous acetonitrile. Phosphopeptides were rehydrated in 30 μL of 50 mM HEPES, pH 8.5 and a 10 μL aliquot of reagent was added. The reaction mixture was incubated at RT for 1 h and then the reaction was quenched with 8 μL of 5% hydroxylamine. TMT sets were then combined, acidified with 20 μL of 20% FA and desalted via C18 SPE. The resultant TMT sets were then fractionated into 6 fractions using a custom capillary LC configuration described previously [30].

### LC–MS/MS analysis

The resulting expression proteomics fractions were separated using a Waters nano-Aquity UPLC system (Waters) equipped with a homemade 75 µm I.D. × 70 cm length C18 column packed with 3-µm Jupiter particles (Phenomenex). A 100-min gradient of 100% mobile phase A (0.1% (v/v) formic acid in water) to 60% (v/v) mobile phase B (0.1% (v/v) FA in acetonitrile) was applied to each fraction. This system was coupled to a Thermo Q-Exactive Plus mass spectrometer for MS/MS analysis. MS Spectra were collected from 300 to 1800 m/z at a mass resolution setting of 70,000. The top 12 most intense ions were selected with an isolation width of 0.7 m/z for higher energy collision dissociation (HCD); + 1 charged species were excluded, and the dynamic exclusion window was 20 s.

Phosphoproteomics fractions were separated as described above, with the gradient length extended to 200 min for each fraction. The UPLC was coupled to a Thermo Q-Exactive HF mass spectrometer for MS analysis using a top 12 DDA method. MS1 and MS2 spectra were collected with a mass resolution of 60 and 30 K, respectively. An isolation window of 2.0 m/z was used for MS2 selection with a dynamic exclusion window of 30 s.

### Data processing and peptide identification

The quantitative TMT LC–MS/MS data were extracted using an approach described elsewhere [27]. The intensities of all TMT reporter ions were extracted using MASIC software [31]. The MS/MS data were preprocessed with DeconMSn [32] and DtaRefinery [33] for recalibration of parent ion m/z. The calibrated spectra were processed with MS-GF+ (v9881) [34], matching against the RefSeq human protein sequence database, release version 37 (https://www.ncbi.nlm.nih.gov/assembly/GCF_000001405.13/), combined with 15 contaminant proteins, including bovine and porcine trypsin and keratins sequences. The only difference with the MS-GF+ search parameters described before was the consideration of TMT 10-plex tags (+ 229.1629 Da) at N-terminus and Lys residues [27]. Expression data were filtered using a minimum peptide length of 6, SpecEValue of < 10E−9.5, and mass measurement accuracy of < 5 ppm. This resulted in a PSM-level FDR of 0.11%, unique peptide sequence FDR of 0.86%, and protein-level FDR of 4.9%. The phosphoproteomic data was filtered using a SpecEValue of < 10E−10 and mass measurement accuracy of < 6 ppm. These cutoffs resulted in a PSM-level FDR of 0.3% and unique peptide FDR of 0.98%.

### Statistical analysis

The TMT reporter intensities were normalized by the reference channel and log2-transformed. Sample-to-sample biases were normalized using the technique described before [27]. To evaluate the effect of storage time, collection site, and TMT plex (batch) and interaction thereof, we applied a linear modeling technique. The analysis was performed using R script [35] and *limma* package [26] of the Bioconductor project [36].

### Pathway-level analysis

We compared estimates of protein abundances from FFPE samples with similar measurements on ovarian high-grade serous carcinoma (HGSC) samples preserved by freezing in optimal cutting temperature (OCT) blocks and used for comprehensive analysis of the HGSC proteome in a previous study [27], to determine whether there was any systematic bias in the ability to identify proteins from FFPE samples. Protein abundance was estimated using spectral counts [37]. When peptides were shared across multiple proteins, the spectral counts were distributed equally across the common proteins (Additional file 3: Table S3). To adjust for differences in total counts between the three datasets the spectral counts per protein were converted to the proportion of all counts within each dataset. For gene set enrichment analysis (GSEA) the protein abundance differences were calculated as the logarithm of the ratios of count proportions. A pathway ontology was constructed from those GO terms containing more than 5 proteins and less than 1000 (to avoid both very narrow and very broad categories). Using these criteria, we retained 8822 GO terms that were represented in the FFPE samples and in two separate analyses of OCT embedded HGSC samples. The significance of the pathway-level difference between the FFPE and OCT samples was computed using GSEA [38]. Specifically, we used implementation of a faster algorithm FGSEA [39] that allowed computation of p-values based on one million permutations.

## Results

### Peptide yield and specimen age

**Fig. 1:**
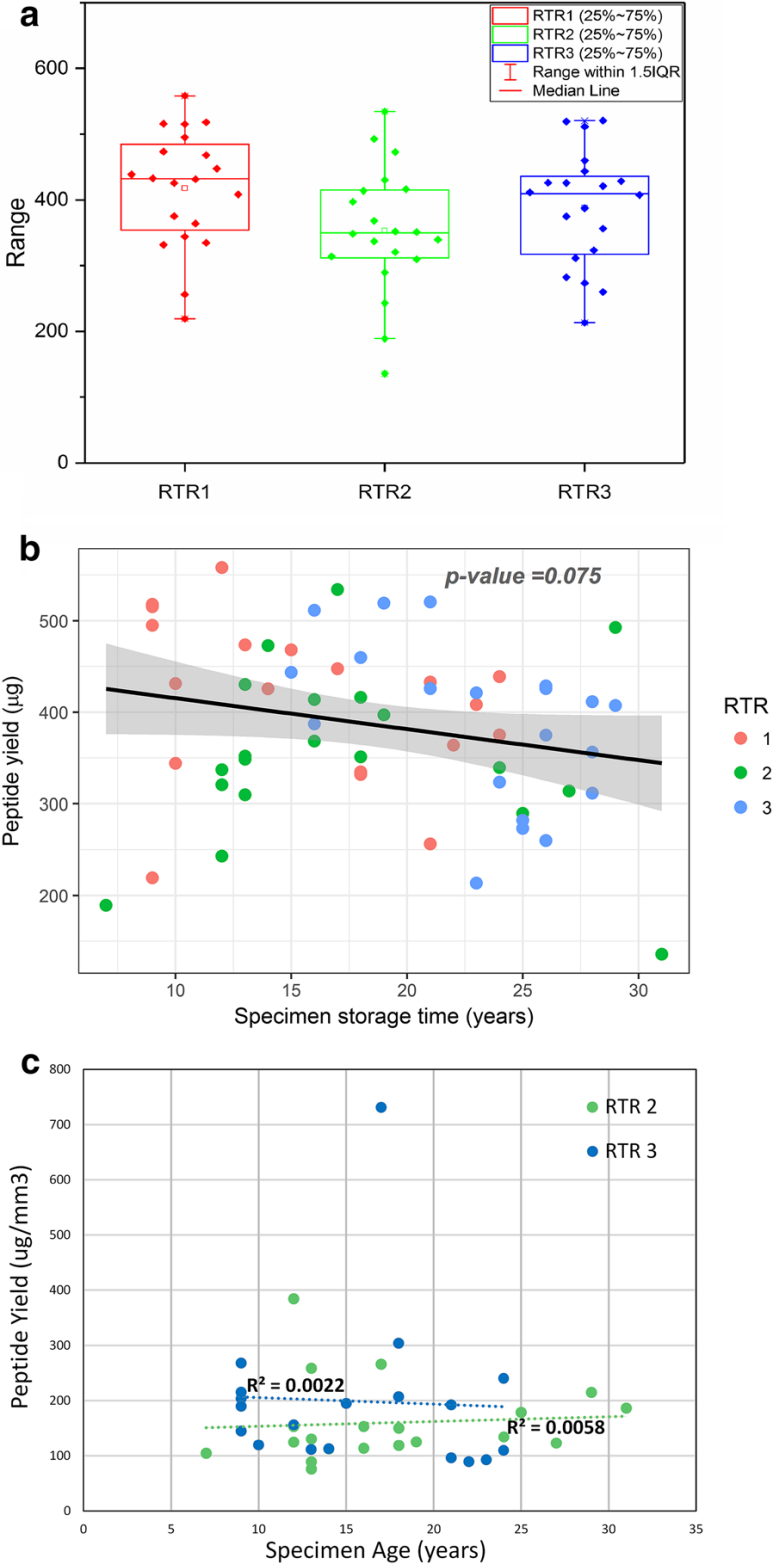
Effect of storage time on peptide yield. **a** Average peptide yield per sample at each RTR. **b** Total peptide extraction yield (µg) from SEER specimens versus specimen age, evaluated by BCA assay. Each point represents total yield from five 10-µm FFPE sections, scraped and pooled in a single tube for sample processing. Different colors are used for each RTR. **c** Peptide yield normalized to tumor volume (surface area × 0.01 mm depth) versus time in storage for the two RTRs reporting tumor dimensions

A total of 60 specimens from three different collection sites were used to evaluate the suitability of SEER specimens for quantitative proteomic analysis. The description of collection sites and specimen age for all samples can be found in Table 1. A more detailed description of the sample cohort can be found in Additional file 4: Table S4. All FFPE samples were extracted and digested using an optimized TFE extraction protocol, and protein yield was evaluated by BCA protein assay. A total of 64,682 peptides covering 8582 proteins and 8073 phosphopeptides from 3089 phosphoproteins were identified and quantified in the SEER sample set (Additional file 5: Table S5). Of these 3089 phosphoproteins, 1620 were from proteins not seen in the expression analysis, Additional file 6: Fig. S1. As shown in Fig. 1, there was no statistically significant difference in peptide yields, either as a function of RTR site (Fig. 1a) or time in storage across all sites (Fig. 1b), whether calculated based on total peptide yield from five, 10 µm thick curls (Fig. 1a, b) or normalized to the reported tumor volume (Fig. 1c). Although peptide yield showed substantial variation, between 135 and 560 µg, it was not statistically associated with time in storage. Thus, it likely reflects other variables such as time in fixative, which could not be controlled in this retrospective study. Most importantly, all samples yielded sufficient protein starting material for expression proteomic analysis, and the results of the proteomic analysis reflected the loading of equal amounts of peptides, independent of the overall yield. The samples were then randomly assigned into TMT 10-plex sets and labeled for analysis. The 18 samples with peptide yields > 400 µg (total in five 10-µm sections) were selected for phosphopeptide analysis. Study design specifics for expression and phosphopeptide analysis are described in Additional file 1: Tables S1 and Additional file 2: Tables S2, respectively.

### Comparison of FFPE and OCT samples

An important QC metric in proteomic analysis is the performance of FFPE tissue samples compared to frozen samples, whether flash-frozen or embedded in OCT. Although there were no matching OCT blocks for the FFPE blocks stored in the SEER registry, our laboratory did participate in both expression and phosphoproteomic analysis of similar HGSC specimens stored by the Cancer Genome Atlas as OCT blocks, with time in storage ranging from 3 to 10 years [27]. Although the procedures and conditions for the current analysis of the SEER FFPE samples and the analysis of The Cancer Genome Atlas (TCGA) OCT specimens were somewhat different (e.g., TMT-10 vs. iTRAQ-4, 300 vs. 200 µg isobarically-labeled peptides for expression proteomics analysis, 3 versus 1.8 mg isobarically-labeled peptides for phosphoproteomics analysis, and Q Exactive versus Velos MS instruments in the SEER and TCGA analysis, respectively, they do share sufficient commonality for qualitative comparisons (e.g., both had the same level of fractionation), Further, there was a significant overlap in the identified proteins, and no bias was seen in molecular weight distribution for the two different analysis, (Additional file 6: Figs. S2 and S3). Although there was no bias in the molecular weight of the proteins identified, at the peptide level the iFFPE samples were enriched for shorter peptides, compared to the OCT samples (Additional file 6: Fig. S4).

**Fig. 2:**
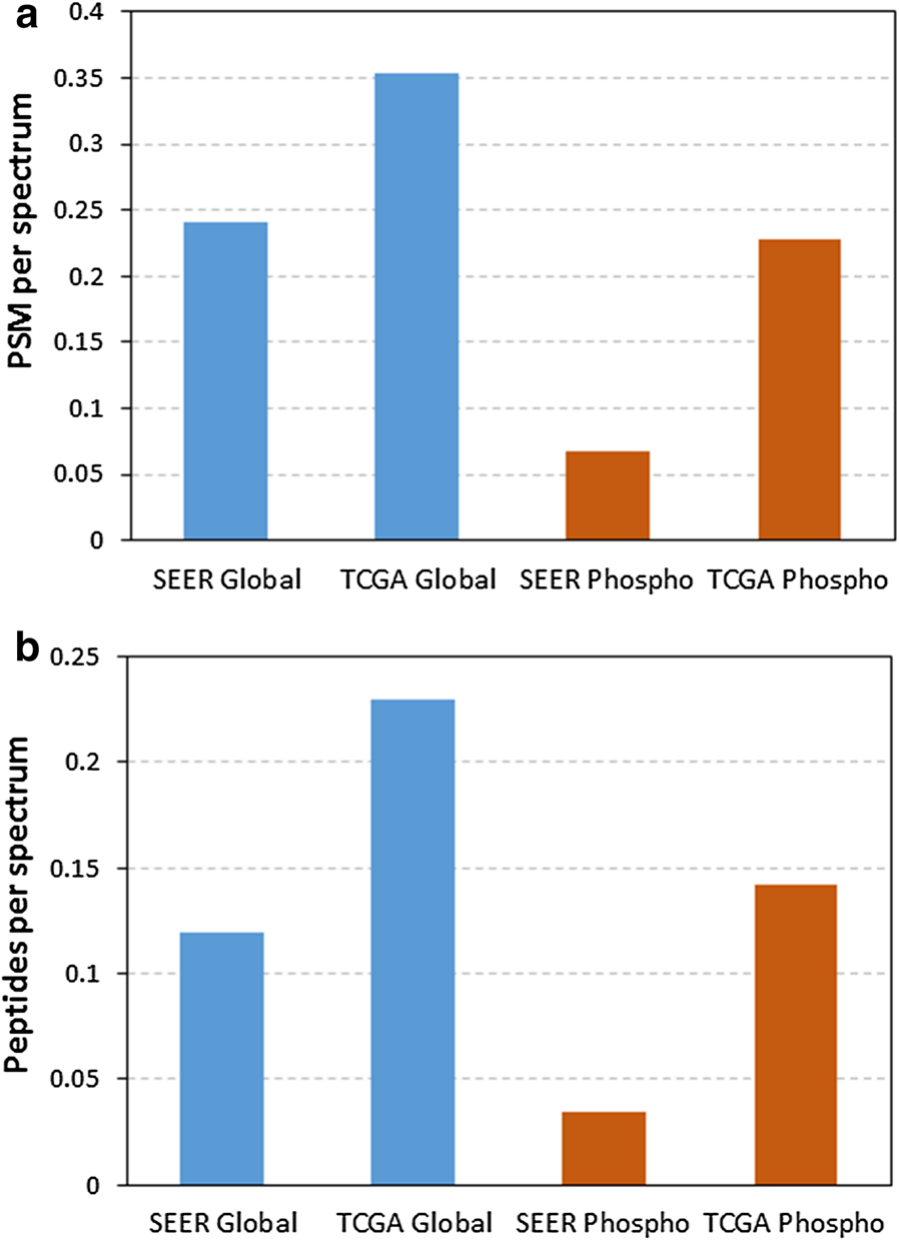
Comparison of FFPE (SEER) and OCT (TCGA) proteome coverage. The comparative analysis was done using both the spectrum identification rates (**a**) and unique peptide identification rates (**b**). The identification rate is calculated as the number of PSM passing a 1% FDR cutoff (**a**) or unique peptide sequences (**b**) divided by the total number of MS/MS spectra taken by the mass spectrometer. The difference in assignment of PSMs and peptides between the TMT-10 (SEER) and iTRAQ-4 (TCGA) labeled samples is also adjusted to account for the known differences as described above

To provide an initial look at the richness of the different sample types we compared the results of the two studies at the MS/MS spectra level. To factor in the difference in data acquisition rate of the different instruments, we used the identification rate instead of the total number of identifications for comparison (i.e., the number of peptide-to-spectrum matches (PSMs) or unique peptide sequences divided by the total number of MS/MS spectra taken by the mass spectrometer). While there have been several studies reporting reduced proteome coverage in TMT analysis compared to iTRAQ-4 analysis [40, 41], the magnitude of change in the different studies was inconsistent, presumably due to the differences in MS instruments and the biological systems in which the tests were performed. In this study, we addressed this by applying correction factors derived from an unpublished NCI study comparing the proteome and phosphoproteome analysis of the same breast tumor tissue samples labeled by TMT-10 and iTRAQ-4, using the same Q Exactive instrument and similar workflow and MS settings as the current study. As illustrated in Fig. 2, peptides and phosphopeptides were identified at significantly higher rate using the OCT specimens from TCGA, compared to the FFPE samples from SEER, at the same level of confidence (FDR < 1% at the protein level), either at the PSM level (A) or unique peptide identification level (B). In the expression proteomic analysis, the unique peptide identification rate is approximately 50% lower in SEER specimens compared to TCGA samples preserved in OCT, corresponding to a 20% reduction in protein identifications. The decrease was more substantial for phosphopeptides, with the FFPE samples yielding about 24% of the identifications obtained from OCT samples.

### Comparison by RTR site

**Fig. 3:**
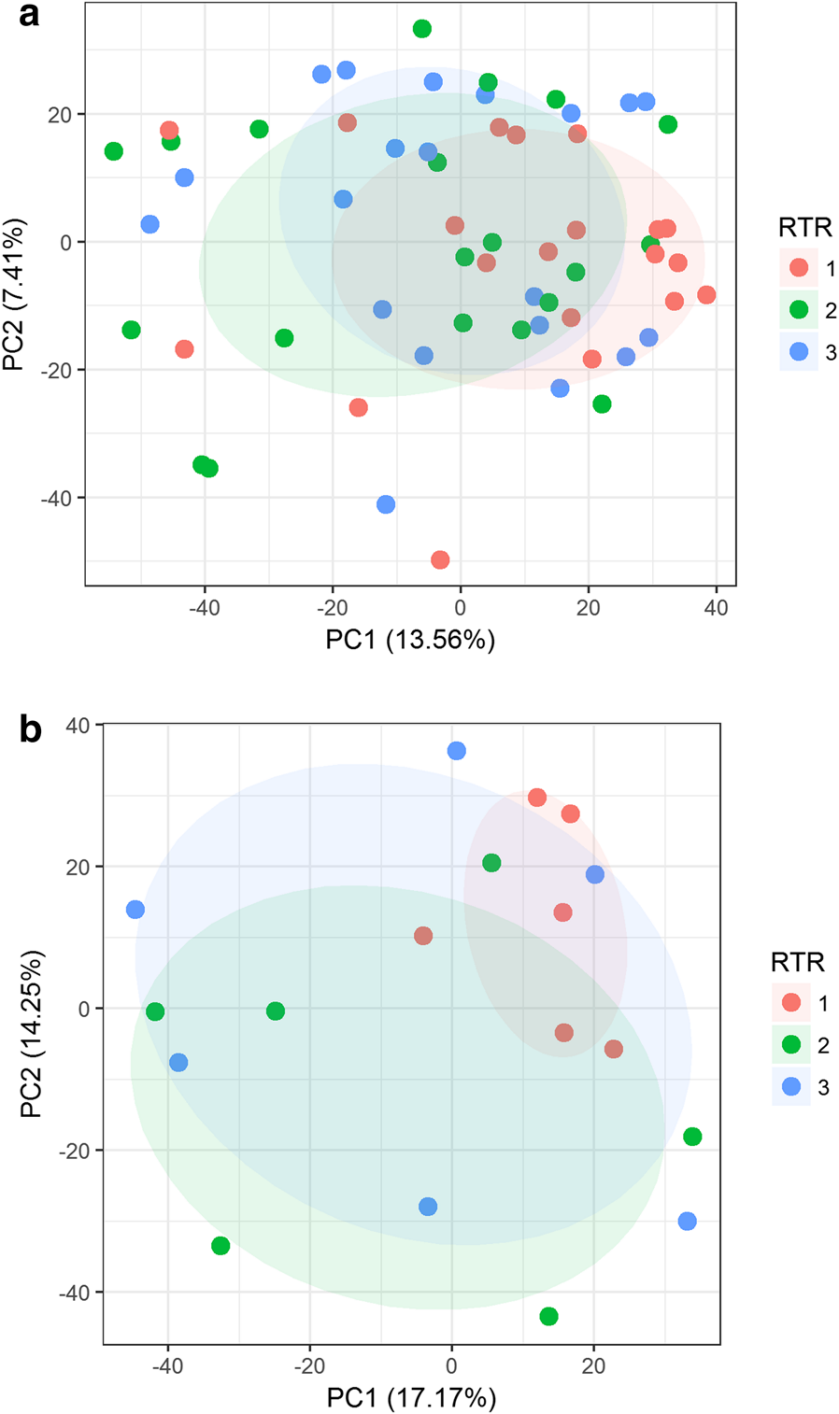
PCA analysis of proteomic results grouped by SEER RTR site: **a** expression proteome results, and **b** phosphoproteome. The lack of any statistically significant effect of RTR on protein abundances within this set of samples was confirmed with ANOVA analysis

To maximize the benefits of analyzing samples from the SEER repository for proteomic and phosphoproteomic analyses, it is important that there be no consistent bias in the results based on the regional RTR where samples were originally collected and subsequently stored. If there is no systematic site bias, proteomic and phosphoproteomic results should be indistinguishable across multiple statistical tests. We used both Principal Component Analysis (PCA) and ANOVA as statistical tests of bias. As illustrated in the PCA plots shown in Fig. 3a, b, neither the expression proteome nor the phosphoproteome showed significant clustering by SEER RTR site. The ANOVA analysis corrected for multiple hypothesis testing showed no statistically significant differences in protein abundance as a function of RTR site (data not shown). As a further demonstration that RTR site is not a significant factor, unsupervised clustering was performed on both expression and phosphoproteomics datasets, Additional file 6: Fig. S5A–D.

### Comparison by specimen time in storage

**Fig. 4:**
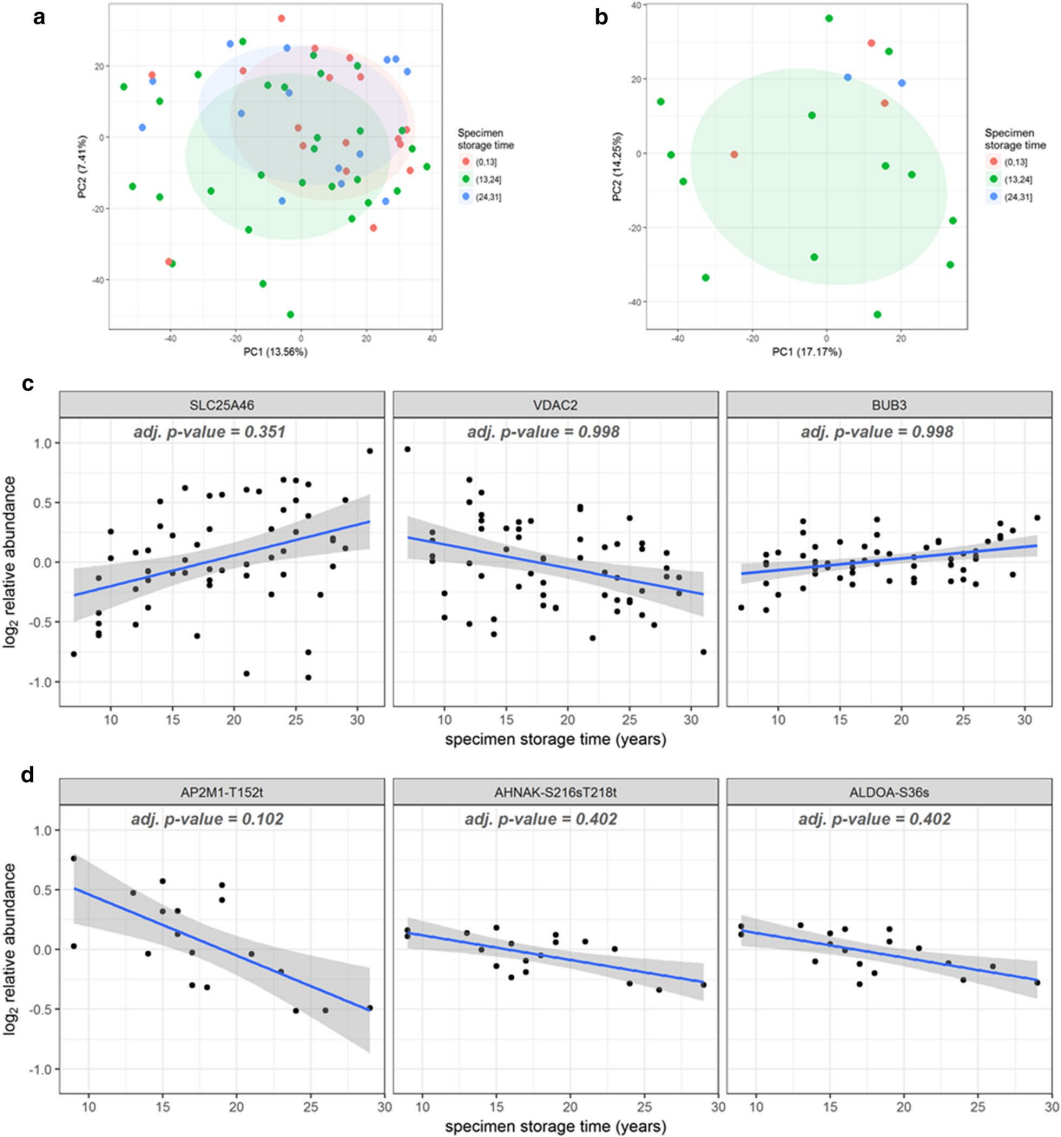
PCA analysis of TMT results analyzed by time in storage: **a** expression protein abundance, and **b** phosphopeptides. Linear regression of relative protein abundance for three proteins with highest significance (**c**) and top 3 most significant phosphosites (**d**)

A substantial feature of the SEER RTR collections is the wide time span represented by the specimens, providing sufficient time for accurate identification of long-term clinical outcomes, assuming adequate patient follow up. However, the utility of this resource requires that protein and phosphoprotein identifications and quantification are stable over time in storage. A PCA analysis of the expression peptide and phosphopeptide results demonstrated no separation of samples based on time in storage, suggesting that the ability to identify specific peptides or phosphopeptides was unaffected by specimen age (Fig. 4a, b, respectively). Equally important is that measurements of relative protein abundance are consistent, independent of time in storage. This question was addressed by plotting the log2 relative abundance as a function of specimen time in storage for the top three most significant proteins (SLC25A46, VDAC2, BUB3) and phosphosites (AP2M1-T152t, AHNAK-S216 sT218t, ALDOA-S36 s) by ANOVA (Fig. 4c, d). The linear model used to test for the effects of storage time included both RTR site and TMT-plex as covariates. After correction for multiple hypothesis testing, there was no statistically significant correlation between storage time and protein abundance. Furthermore, Fig. 4c demonstrates that biological variability is greater than the variability associated with storage time, indicating that storage time is not a significant confounding variable for analysis of protein abundance.

### Testing for differences in GO term representation

**Fig. 5:**
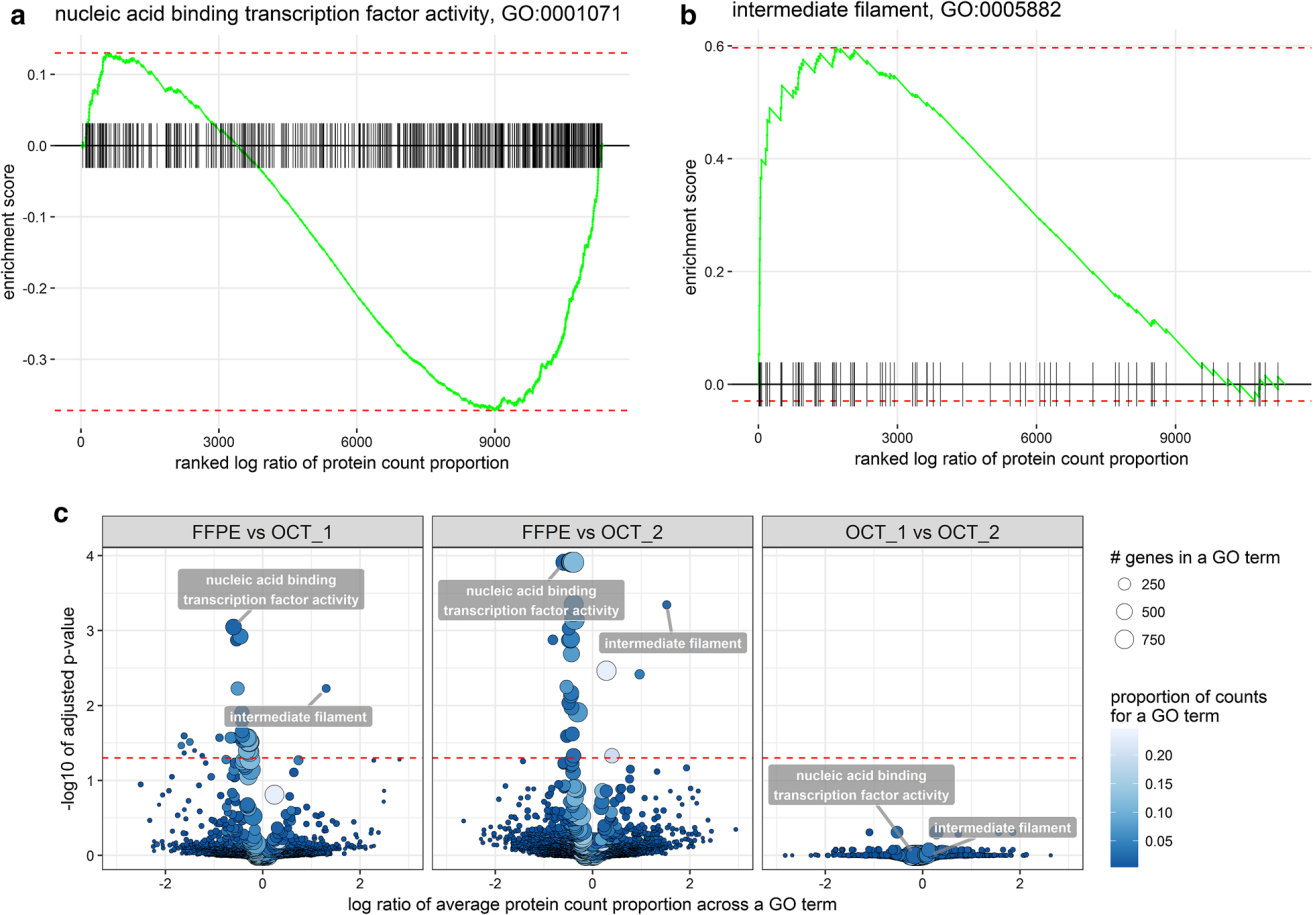
Comparison of GSEA enrichment in FFPE versus OCT samples. **a** and **b** are examples of the GSEA plots for the most depleted and enriched GO terms comparing FFPE to OCT. The significance of the test depends on the degree of concordance between changes in protein abundance within a GO term. Particular groupings of proteins at the low and high ends of the ranked list indicated non-random depletion and enrichment of the corresponding GO terms. The significance of the enrichment is based on one million permutations which were computed using FGSEA R package. **c** is the volcano plot reflecting significance and estimate fold of change of the GO term. The list of significantly affected GO terms is available in the Additional file 7: Table S6. The pattern and most significant GO terms remained consistent when FFPE was compared to OCT data, independent of the OCT analysis site (PNNL vs. JHU). Noteworthy, comparing two OCT datasets as negative control test set yielded no significantly different GO terms (right panel), thus demonstrating the validity of the statistical test

Interpretation of proteomic results from FFPE specimens requires some knowledge of any functional pathways that are disproportionately affected by formalin fixation. To identify any protein subcategories preferentially lost during fixation, we applied GSEA analysis to GO-term level measurements of protein abundance within each sample type, defined as proportion of total spectral counts. Spectral counts from either TMT-10 or iTRAQ-4 analysis provide an averaged representation of protein abundance in each multiplexed experiment, instead of that in the individual samples, and thus is well-suited for the comparison of within-sample protein changes across the two sample types. We used an experimental design that allowed us to compare HGSC samples processed from FFPE versus OCT, as well as overlapping HGSC samples in OCT processed in two distinct laboratories; the PNNL and JHU analyses of the TCGA HGSC samples embedded in OCT [27]. The comparison of OCT samples analyzed at PNNL and JHU serves as a negative control, since the samples themselves, and thus the embedding process, were identical. The left and middle panels in Fig. 5c showed the comparisons between FFPE samples and OCT samples from JHU (40 significant GO terms) and PNNL (38 significant GO terms) datasets, respectively. As expected, there were no significant differences in matched OCT samples analyzed in two different facilities using the same workflow (Fig. 5c, right panel). In contrast, there were a number of significant differences in enriched GO terms between FFPE samples and OCT samples, regardless of the analysis site. Interestingly, one of the most consistently affected GO categories was DNA-binding proteins, suggesting that cross-linking of proteins and DNA in FFPE samples significantly impaired peptide identification and quantitation by MS. However, because FFPE samples require a different protein extraction method compared to OCT, we cannot distinguish between the impacts of extraction method and FFPE preservation. As a control for nuclear localization, independent of DNA binding, we specifically examined the retrieval of nuclear envelope proteins (GO:0005635). The enrichment was clearly non-significant (Additional file 6: Fig. S6) with p values adjusted for multiple hypothesis testing of 0.52 for SEER versus OCT1 and 0.82 for SEER versus OCT2. This suggests that nuclear localization itself is less significant than other factors, such as the formalin-enhanced crosslinking of surface accessible lysine residues to negatively charged DNA in DNA-binding proteins.

## Discussion

Despite the acceptable peptide yields, the number of identified peptides in FFPE samples was approximately 50–60% lower, corresponding to a 20% reduction in identifications at the protein level, compared to identifications from OCT embedded samples representing the same tumor type (HGSC), and stored for similar lengths of time. This is consistent with literature reports that useful expression proteomics data is obtainable from FFPE specimens [9, 12]. A comparison of FFPE and fresh frozen tissue carried out by Ostasiewicz et al., found that similar depths of coverage were obtained for expression proteomics when using a filter aided sample preparation (FASP) approach [22]. However, this study was carried out using a label-free approach on recently preserved specimens, and did not profile to the depth of this investigation. In the current study, the observed decrease was more significant for phosphopeptides (~ 20% of the OCT identifications) than for unmodified peptides, potentially limiting the use of FFPE samples for phosphoproteomics, but not for expression proteomics. In the Ostasiewicz investigation, they found that they were able to obtain similar depth of coverage for the phosphoproteome when using FASP in combination with a label-free approach. This finding suggests that further refinements to the approach here will yield improvements to the phosphoproteomics results. To the best of our knowledge, the current study represents the deepest reported proteome and phosphoproteome coverage obtained with isobaric labeling of FFPE specimens [40, 41]. The ability to detect 4000–4500 unique proteins from five pooled FFPE sections, yielding an average of 380 µg of protein, represents an adequate depth of coverage for most proteomic studies. Our analysis identified a number of disease associated proteins including MUC16, WFDC2, SPP1, MSLN, SPON1, FOLR1, and TP53. Furthermore, phosphorylation sites were detected on SPP1, MSLN and TP53. If deeper coverage is required, further optimization may be necessary to increase the identification of lower abundance peptides and proteins.

A concern in the use of FFPE specimens is the toxicity associated with the use of xylenes to deparaffinize tissue blocks. Although there are methods reported in the literature that aim to eliminate the use of xylene for deparaffinization [42, 43], these methods have had very limited application to LC–MS/MS based proteomics and often use reagents that are incompatible with mass spectrometry, such as surfactants and mineral oil. Because the SEER samples were prepared with xylene, and because of uncertainties regarding the use of xylene substitutes for LC–MS/MS, xylene alternatives were not employed here to avoid confounding factors in our evaluation of the SEER specimens.

Although the high degree of biological variability precludes any conclusions about storage time-dependent changes, it would appear that the observed changes occur rapidly after initial fixation and remain relatively stable thereafter, consistent with the hypothesis that artefacts induced by formalin fixation process are the major contributor to decreased recovery. As further evidence that formalin-induced chemical cross-linking is responsible for most of the observed differences between FFPE and OCT processed specimens, the FFPE samples appeared to be under-represented in DNA-binding proteins, but not in general nuclear envelope proteins. Since formalin treatment is known to induce DNA-protein cross-links [17, 44], it is likely that cross-linking to nucleic acids interfered with peptide identification and quantitation.

In summary, this study demonstrates that the FFPE specimens from the SEER registry can be used for quantitative proteomic analysis. Accordingly, these collections represent a significant potential resource for hypothesis-driven cancer research. Our analysis demonstrates that, after an initial decline in ‘identifiable’ peptides and phosphopeptides due to the formalin fixation process, there is no further significant degradation incurred with increasing storage time to the limits of this study (32 years). Furthermore, there were no statistically significant differences observed between collection sites, indicating that sample cohorts can be constructed from multiple collection sites. Sufficient protein was obtained from each specimen in the study for expression analysis, while 1/3 of specimens yielded sufficient peptide mass for phosphopeptide analysis. However, further optimization of sample handling and data analysis tailored to FFPE samples could further mitigate the detrimental impacts of fixation.

## Conclusions

Given the rich clinical outcomes data available, the useful levels of peptide recovery, and successful MS-based analysis observed for SEER RTR specimens, we conclude that archival FFPE specimens are a valuable resource for expression proteomic experiments. These specimens offer an exciting opportunity for researchers to interrogate the statistical association between protein abundance and clinical outcome; however, certain functional categories (e.g., DNA-binding proteins and phosphopeptides) may be under-represented in the processed data set, presumably due to artefacts of fixation.

Although questions remain about the biological relevance of phosphorylated peptides after fixation, numerous reports in the literature suggest this modification is well preserved by fixation [22–25, 45]. While discoveries based on FFPE specimens should ideally be validated using flash frozen or OCT embedded specimens, the use of residual FFPE specimens for initial discovery and verification experiments can significantly reduce the need for flash frozen or OCT embedded specimens, and significantly expand the cohorts available for retrospective analysis.

## Additional files

**Additional file 1: Table S1.**  TMT labeling scheme for expression proteomics.

**Additional file 2: Table S2.**  TMT labeling scheme for phosphoproteomics.

**Additional file 3: Table S3.** Weighted spectral count table used for GSEA.

**Additional file 4: Table S4.**  Table of specimen age and RTR.

**Additional file 5: Table S5.**  Identified peptides, proteins, and phosphopeptides.

**Additional file 6.**  Supplementary figures.

**Additional file 7: Table S6.** Significantly affected GO terms.

## Abbreviations

NCI: National Cancer Institute
PNNL: Pacific Northwest National Laboratory
OCT: optimal cutting temperature
FFPE: formalin fixed, paraffin embedded
SEER: surveillance, epidemiology and end results
RTR: residual tissue repository
NGS: next-generation sequencing
WES: whole exome sequencing
TMT: tandem mass tags
LC–MS: liquid chromatography–mass spectrometry
MS/MS: tandem mass spectrometry
TFE: 2,2,2-trifluoroethanol
IMAC: immobilized metal affinity chromatography
UPLC: ultra performance liquid chromatography
MS-GF: mass spectrometry generating function
HGSC: high-grade serous carcinoma
GSEA: gene set enrichment analysis
TCGA: the cancer genome atlas
PSM: peptide-to-spectrum match
iTRAQ: isobaric tag for relative and absolute quantitation
ANOVA: analysis of variation
PCA: principle component analysis
GO: gene ontology
JHU: Johns Hopkins University
FASP: filter aided sample preparation
