# Supplementary material for: Residual tissue repositories as a resource for population-based cancer proteomic studies
Source: Clin Proteomics. 2018 Aug 3;15:26. doi: 10.1186/s12014-018-9202-4 (PMC6074037; doi:10.1186/s12014-018-9202-4)
Supplement: Supplementary file 6 — Additional file 6. Supplementary figures. [file 12014_2018_9202_MOESM6_ESM.pdf]

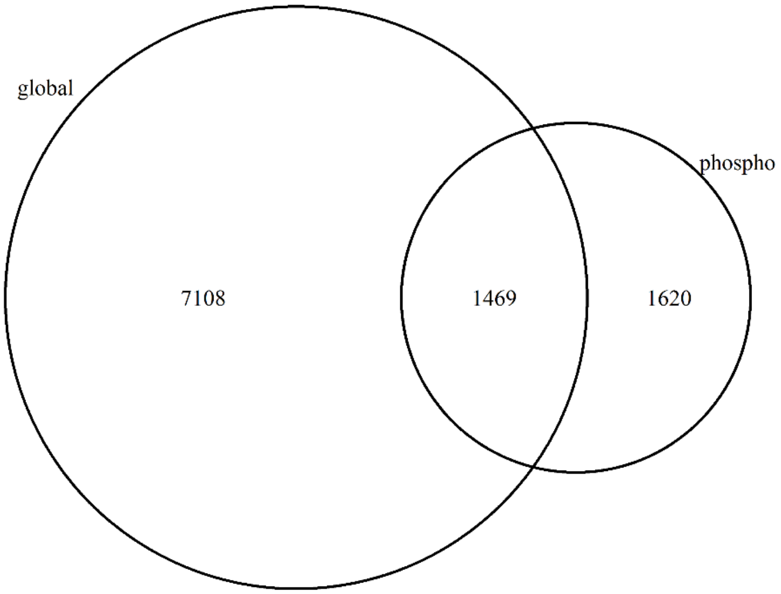

Fig. S1. Venn Diagram showing the overlap of protein counts at the gene level between the expression (global) proteomics and the phosphoproteomic analysis of SEER FFPE tissues.

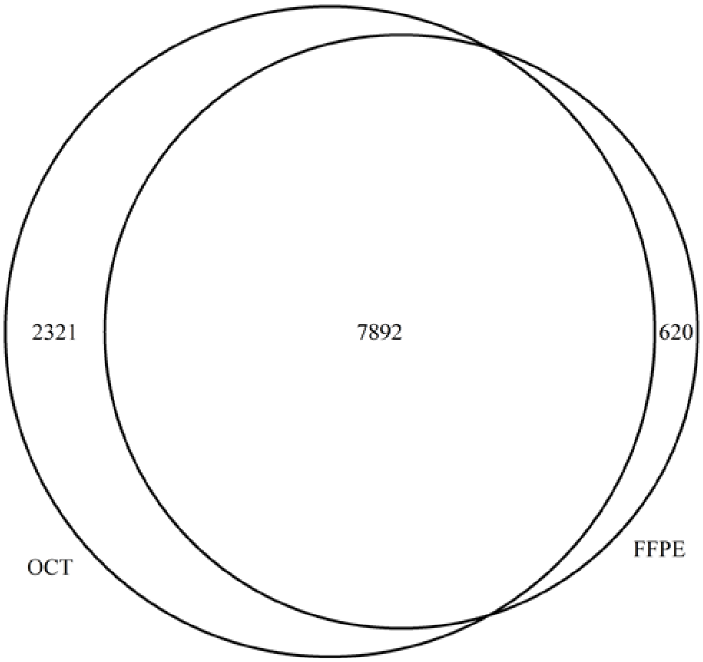

Fig. S2. Venn Diagram showing the overlap of protein counts at the gene level between the PNNL OCT and SEER FFPE tissue analyses.

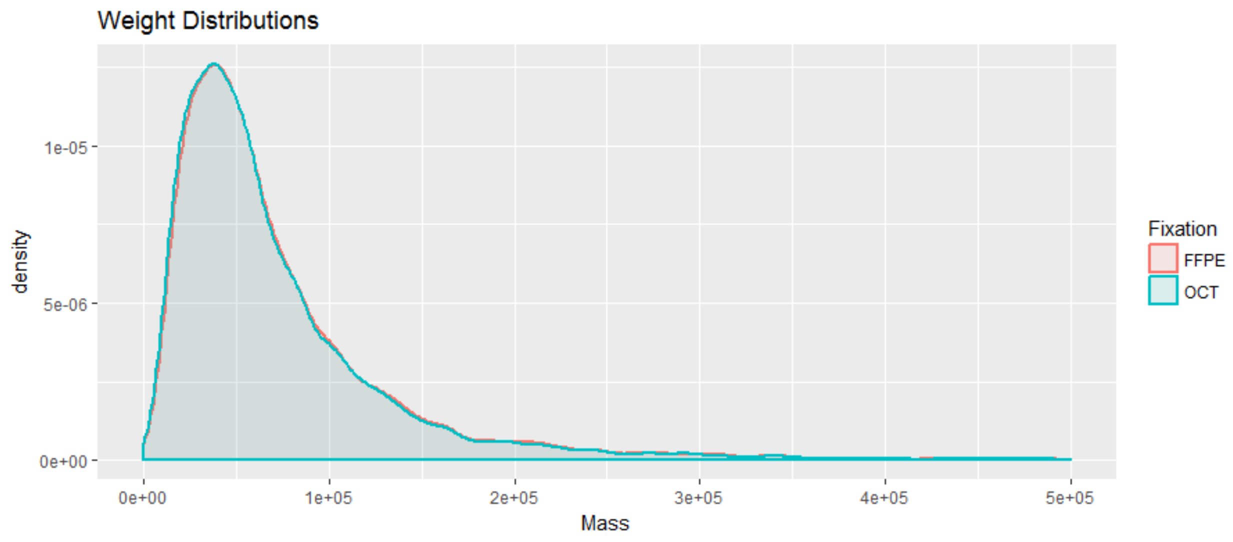

Fig. S3. Density plot overlay of protein MW for proteins identified in the PNNL OCT and SEER FFPE datasets.

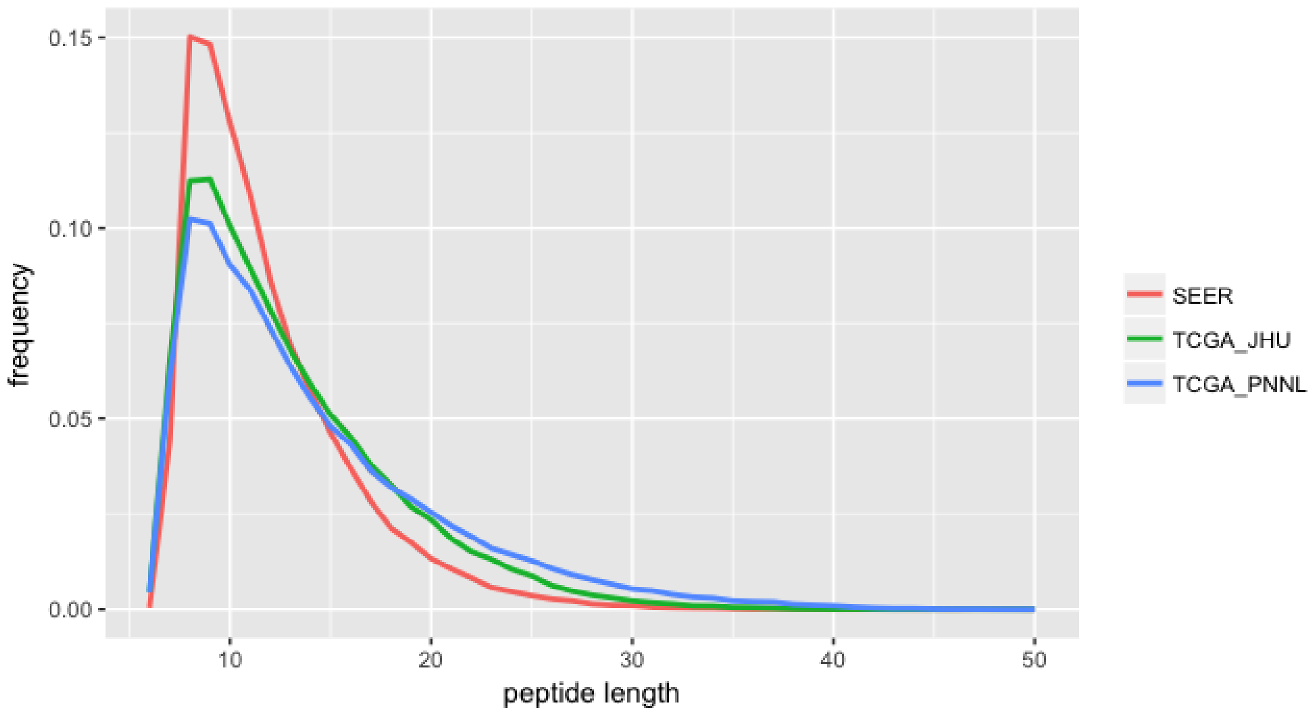

Fig. S4. Density plot overlay of peptide length in the PNNL and JHU OCT studies, and SEER FFPE datasets.

Unsupervised Clustering Analyses (Supplementary Figures 5A-D)

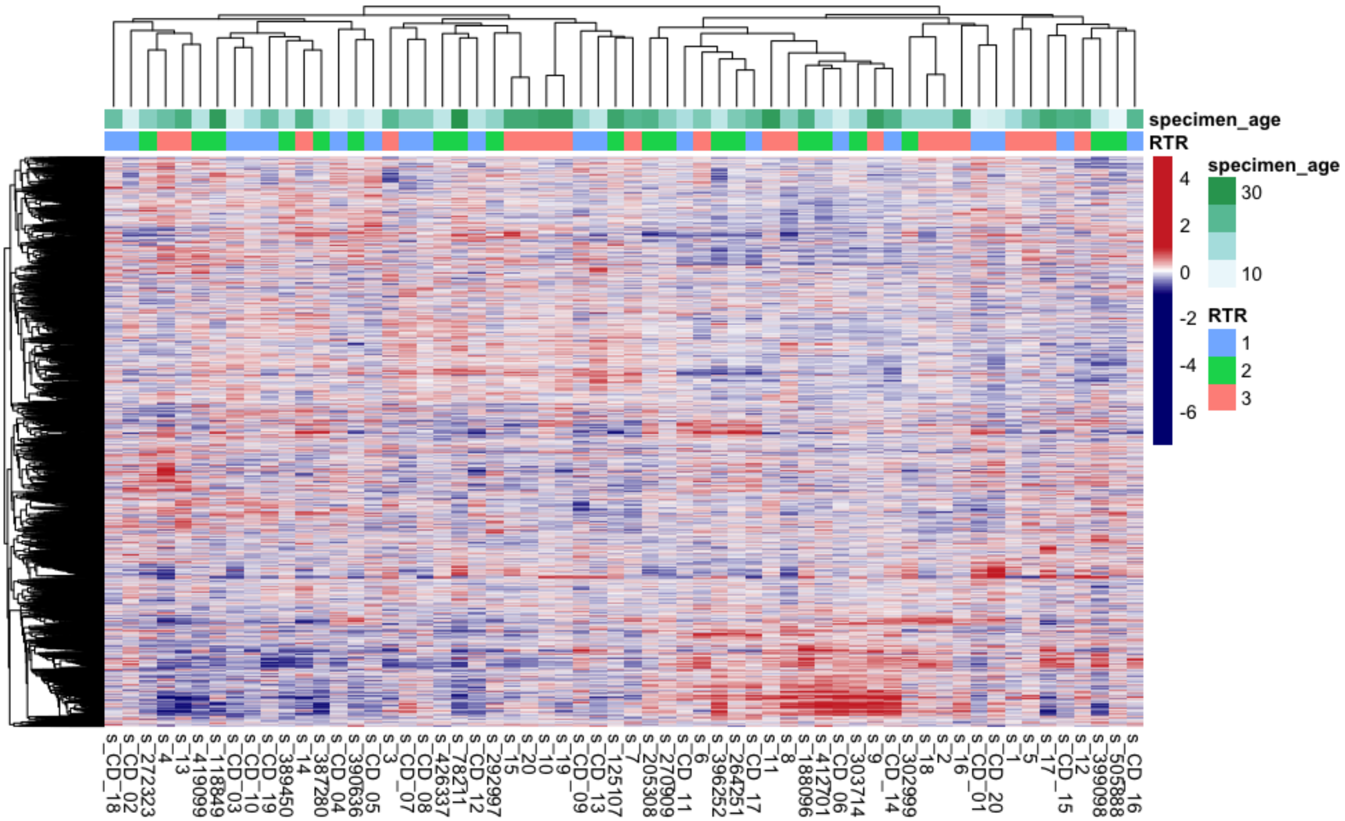

Fig. S5A. Unsupervised Clustering of Global Expression Proteomics (Full dataset).

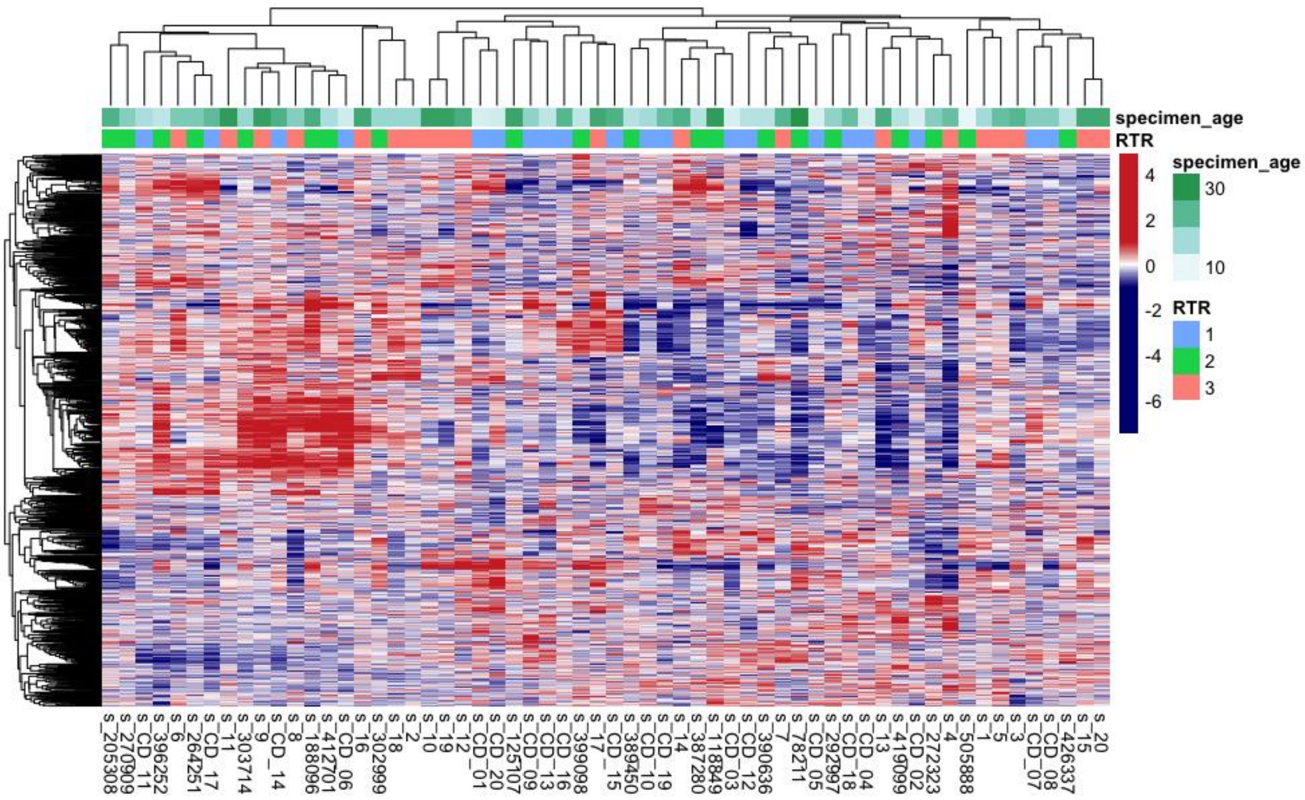

Fig. S5B. Unsupervised Clustering of Global Expression Proteomics (Most Variable 25%).

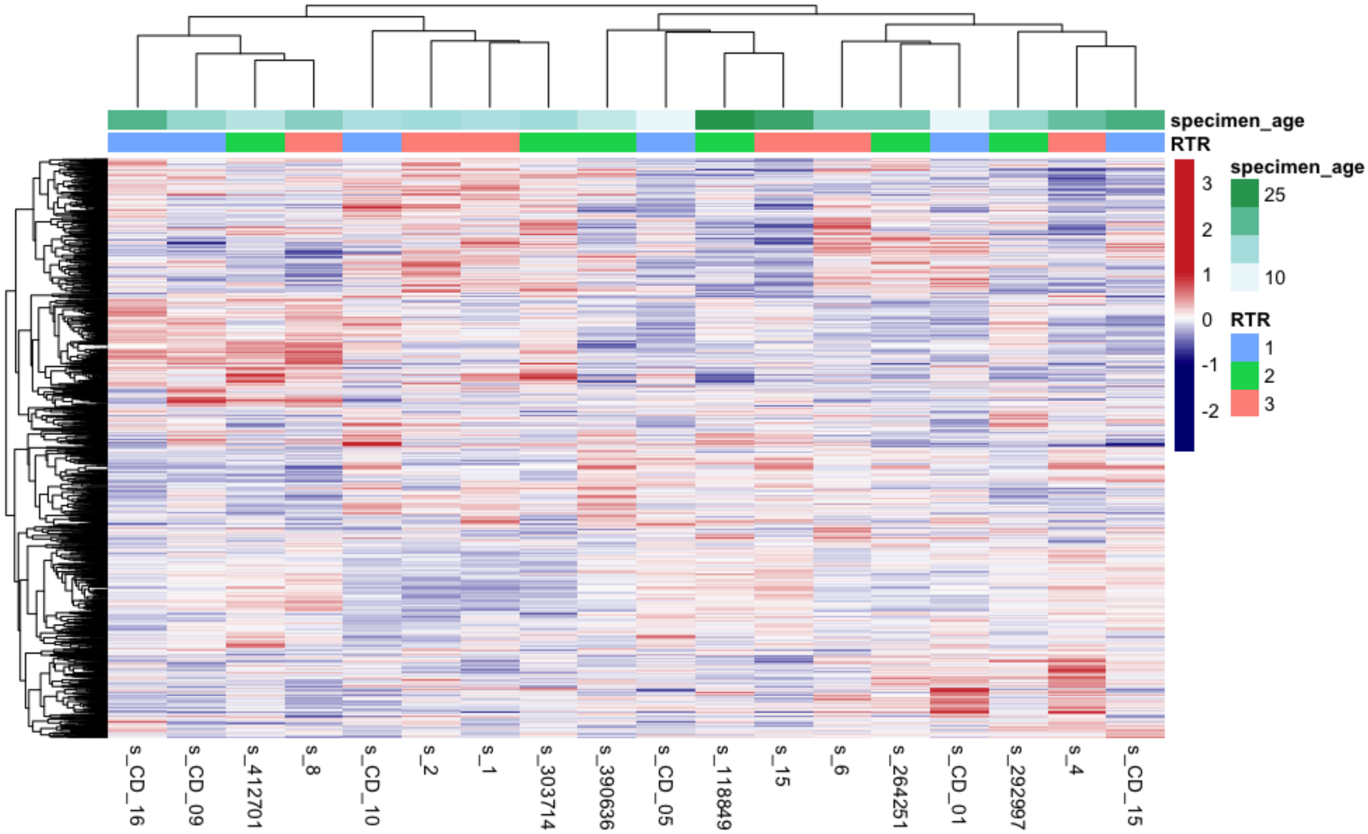

Fig. S5C. Unsupervised Clustering of Phosphoproteomics (Full Dataset).

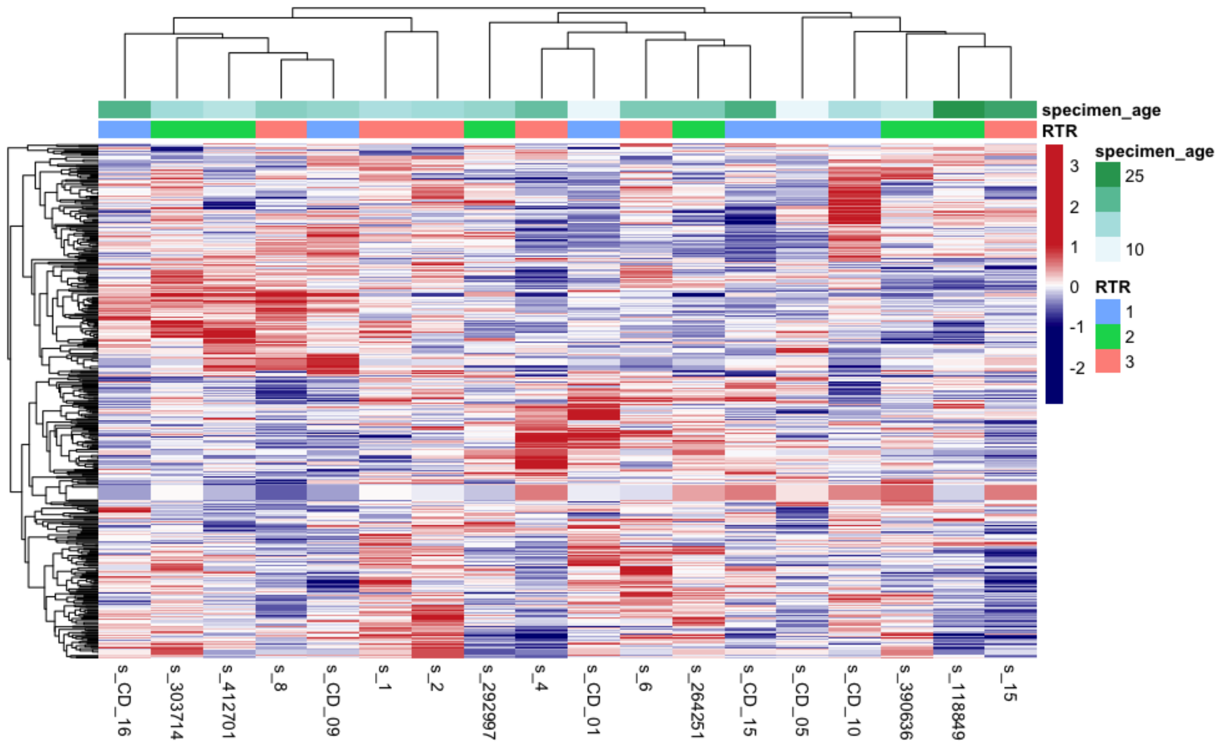

Fig. S5D. Unsupervised Clustering of Phosphoproteomics (Most Variable 25%).

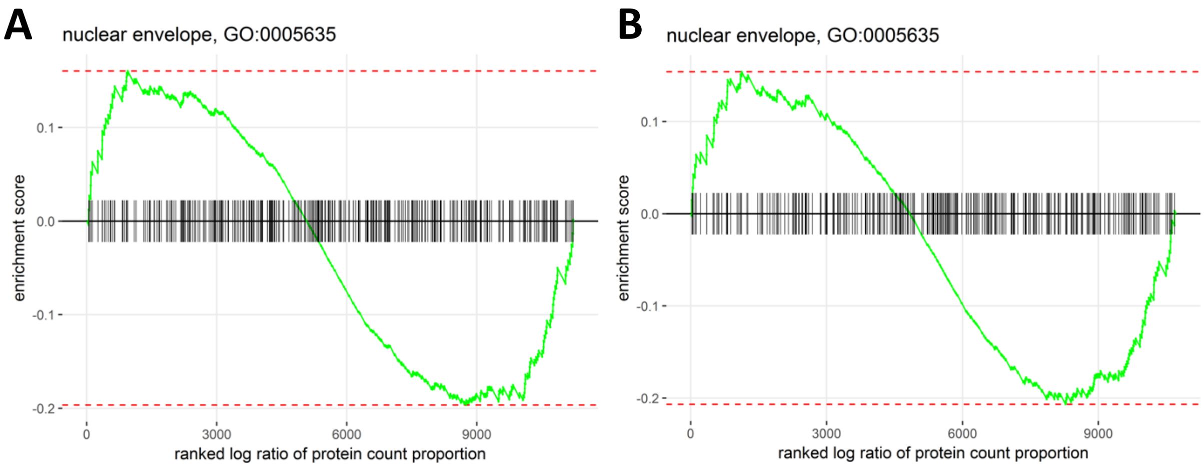

Fig. S6. GSEA test results for nuclear envelope GO:0005635 term. The broad terms contains 353 measured proteins that are part of nuclear membrane, pore, nucleoplasmic reticulum and other nuclear components that exclude DNA-binding proteins. **A** and **B** are comparison of SEER vs OCT1 and OCT2 datasets respectively. The p-values are 0.99 and 0.94 correspondingly.
